# A Case of Radiation Fibrosis Appearing as Mass-Like Consolidation after SBRT with Elevation of Serum CEA

**DOI:** 10.1155/2010/986706

**Published:** 2010-06-06

**Authors:** Kotaro Terashima, Yoshiyuki Shioyama, Satoshi Nomoto, Saiji Ohga, Takeshi Nonoshita, Kayoko Ohnishi, Kazushige Atsumi, Hidetake Yabuuchi, Hideki Hirata, Hiroshi Honda

**Affiliations:** ^1^Department of Clinical Radiology, Graduate School of Medical Sciences, Kyushu University, Fukuoka 812-8582, Japan; ^2^Department of Radiation Technology, School of Health Sciences, Faculty of Medicine, Kyushu University, Fukuoka 812-8582, Japan

## Abstract

We report a case of radiation fibrosis appearing as mass-like consolidation, which was difficult to distinguish from local recurrence. A 72-year-old woman was diagnosed as having primary lung cancer (cT1N0M0 stage IA) in the right upper lobe and was treated with SBRT of 48 Gy in 4 fractions. After 12 months, mass-like consolidation appeared around the irradiated area, and after 13 months, it had increased in size. FDG-PET revealed high uptake (SUV max = 5.61) for the consolidation. CT-guided biopsy was performed, but we could not confirm the diagnosis. Considering her poor respiratory function and her age, short-interval follow-up was performed. After 15 months, the consolidation enlarged at the dorsal side, and carcinoembryonic antigen (CEA) became elevated (14.6 ng/mL). Serum KL-6 (436 U/mL) and SP-D (204 ng/mL) were also elevated. However, after 16 months, serum CEA slightly decreased. The consolidation gradually retracted on follow-up CT images. CEA, KL-6, and SP-D were also decreased by degrees. After 40 months, there is no evidence of local recurrence.

## 1. Introduction

Stereotactic body radiotherapy (SBRT) is an effective therapy for early-stage lung cancer. In some cases after SBRT, dense consolidation is observed over or around the tumors on follow-up computed tomography (CT). Sometimes, it appears as mass-like consolidation, in which case it is difficult to distinguish radiation fibrosis from local recurrence, though it is important from a clinical point of view to make this distinction. 

We experienced a case of radiation fibrosis appearing as mass-like consolidation with elevation of serum carcinoembryonic antigen (CEA), which was difficult to distinguish from local recurrence. In this paper, we present this case along with a review of the literature.

## 2. Case Report

**Figure 1:**
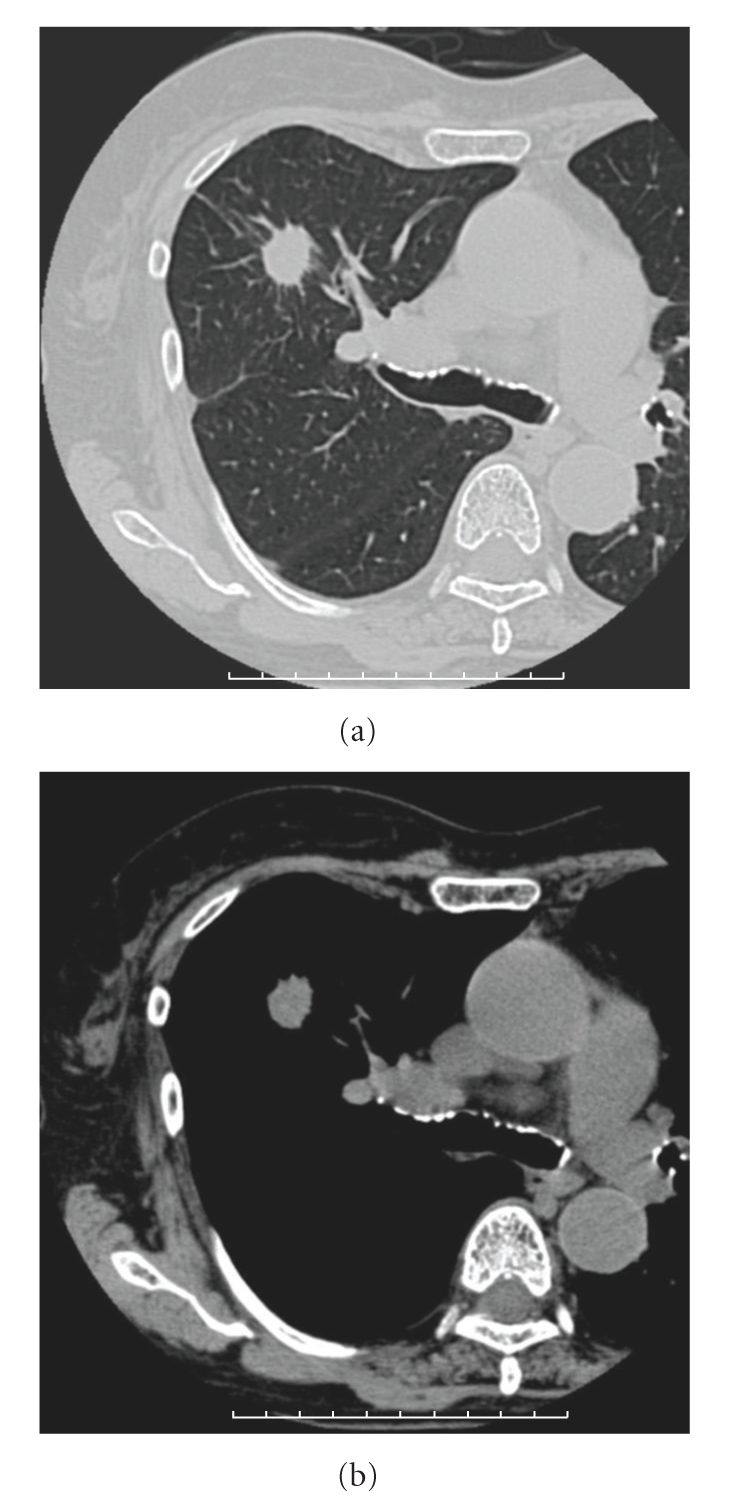
CT images before SBRT showing a solitary lung tumor in the right upper lobe.

**Figure 2:**
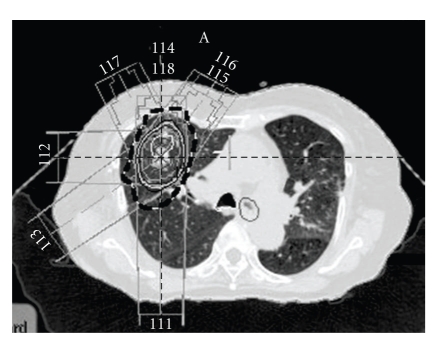
Dose distribution of treatment plan. The dashed line indicates the 50% isodose curve.

A 72-year-old woman was diagnosed as having primary lung cancer (cT1N0M0 stage*Ⅰ*A) of the right upper lobe (Figure 1). Because her respiratory function was not sufficient for her to undergo surgical resection, she was treated with SBRT. Irradiation was performed with a 4-MV and 10-MV linear accelerator. Forty-eight Gy in 4 fractions was prescribed at the isocenter with multiple static ports. The isodose distribution of SBRT is shown in Figure 2. Serum CEA was elevated to 14.7 n/mL, but serum KL-6 (230 U/mL) and SP-D (72.8 ng/mL) were not elevated before SBRT. She was a heavy smoker, and her Brinkman index was 900 (20 × 45 years).

**Figure 3:**
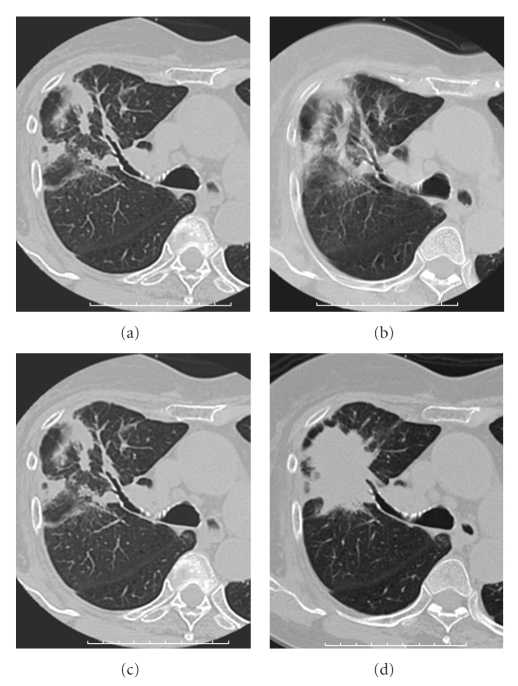
Serial CT images after SBRT. (a), (b), (c) CT images at 2, 6, and 9 months after SBRT show irregularly shaped patchy consolidation in the irradiated area. (d) CT images at 12 months after SBRT showing the appearance of mass-like consolidation in the irradiated area.

**Figure 4:**
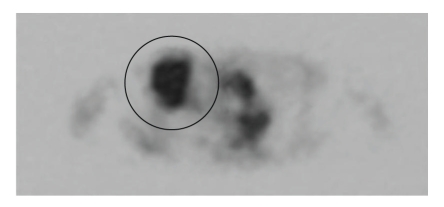
FDG-PET at 13 months after SBRT. High FDG uptake (SUVmax  = 5.61) was observed for the consolidation.

Follow-up CT scans were performed 2, 6, 9, and 12 months after SBRT. On CT images 2 months after treatment, irregularly shaped patchy consolidation, which was considered radiation pneumonitis, appeared in the irradiated field (Figure 3(a)). After 6 and 9 months, the patchy consolidation was retracted, which was considered to be typical radiation fibrosis (Figures 3(b), 3(c)), and serum CEA gradually decreased. However, after 12 months, mass-like consolidation appeared around the irradiated area (Figure 3(d)), and after 13 months, it had increased in size. Because ecstatic air-containing bronchi were not observed within the consolidation, and the 3b branch of the right lung was displaced on CT images, the possibility of local recurrence was considered. At this time, serum CEA, KL-6, and SP-D were not elevated compared with the previous values. FDG-PET revealed high uptake (SUVmax  = 5.61) for the consolidation (Figure 4). CT-guided biopsy was performed. The result of cytology was class II, and histologically cancer cells were not detected. Because there was no definite evidence of malignancy, and considering her poor respiratory function and her age, short-interval follow-up was performed.

**Figure 5:**
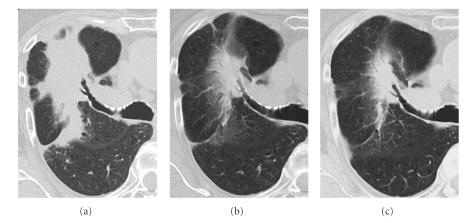
Serial CT images. (a) CT images at 15 months after SBRT show the mass-like consolidation enlarged at its dorsal side. (b), (c) CT images at 18 and 27 months after SBRT show that the mass-like consolidation gradually retracted.

**Figure 6:**
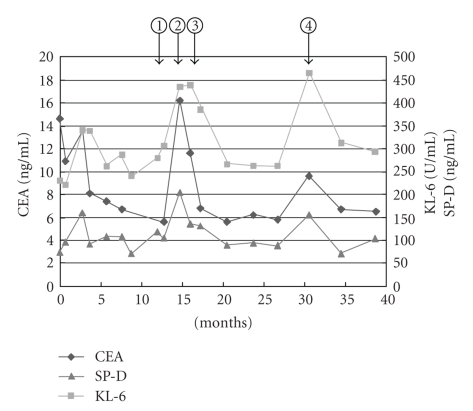
Serial changes of serum CEA, KL-6, and SP-D. (1) At 12 months after SBRT, mass-like consolidation appeared in the irradiated area. (2) At 15 months, the mass-like consolidation enlarged at the dorsal side and serum CEA increased accompanied by KL-6 and SP-D. (3) At 16 months, serum CEA was slightly decreased. (4) At 31 months, minimal lung opacities suggesting pneumonia in the left lower lobe were observed on CT images, but no significant change was seen in the irradiated area.

After 15 months, the consolidation enlarged at the dorsal side, and serum CEA was elevated to 14.6 ng/mL. Serum KL-6 and SP-D were also elevated to 436 U/mL and 204 ng/mL, respectively. However, after 16 months, serum CEA started to decrease slightly. Furthermore, the consolidation gradually retracted on follow-up CT images (Figure 5). CEA, KL-6, and SP-D decreased by degrees (Figure 6), although transient re-elevation was observed after 31 months, accompanied by the development of pneumonia in the left lower lung. At 40 months after the completion of SBRT, she is alive without local recurrence.

## 3. Discussion

On CT images, parenchymal consolidation with a straight lateral margin and air bronchograms is typical for radiation fibrosis, whereas a homogeneous opacity without air bronchograms and with a convex border is strongly suggestive of recurrent tumor in the irradiated lung [1]. In addition, the filling in of bronchi within radiation fibrosis is abnormal and usually represents local recurrent malignancy or a superimposed infection [2]. Koenig et al. firstly described as mass-like radiation fibrosis related to 3-D conformal radiotherapy [3]. Mass-like radiation fibrosis in their cases was accompanied by volume loss and bronchiectasis. However, in our case, neither volume loss nor bronchiectasis was observed at the presence of mass-like consolidation. Therefore, it was difficult to differentially diagnose this mass-like consolidation as radiation fibrosis from local recurrence. Aoki et al. [4] reported patchy consolidation or discrete consolidation in 74% of cases during the first 6 months after SBRT. In SBRT, the shape of the dose distribution with a lower dose tended to become large and irregular, while a higher dose could be concentrated uniformly on the tumor. Matsuo et al. [5] reported that mass-like consolidation appeared in 27 (68%) of 40 tumors treated with SBRT. Of these 27 mass-like consolidations, 24 were radiation-induced lung injuries (RILI) and 3 were local recurrences. According to the previous reports mentioned above, it is not uncommon for mass-like consolidation to appear after SBRT. In conventional radiation therapy, the shape of the dose distribution of irradiated lungs is simple, and the boundary between the nonirradiated and irradiated lung is usually distinct [4]. In contrast, in SBRT, multiple non coplanar portals with various directions were used. And the dose distribution usually has a 3-D shape and concentrates around the tumor. Therefore, radiation fibrosis after SBRT can appear in a 3-D shape, and is sometimes observed as mass-like lesions. In our case, also, the consolidation after SBRT had a 3-D shape, and was observed in approximately 50% or more of the isodose area.

Matsuo et al. [5] also reported that mass-like consolidations appeared at a median of 5 months (range, 2 to 9 months) after SBRT. The time to the appearance of the mass-like consolidations after SBRT was 2 to 9 months (median, 5 months) in RILI cases and 4 to 7 months (median, 7 months) in local recurrence cases. There was no significant difference in the time to appearance between RILI and local recurrence. According to their report, the size of the mass-like consolidations did not increase in any RILI cases after 12 months or later. In our case, however, the mass-like consolidation appeared at 12 months after SBRT, and had increased in size at 13 months. Radiation fibrosis can appear as mass-like consolidation even 12 months or more after the completion of SBRT.

In our case, serum CEA was also elevated in addition to the mass-like consolidation. The CEA elevation and the late appearance of the mass-like consolidation made it very difficult to differentially diagnose this condition as RILI from local recurrence. Several authors have reported that CEA elevation is observed in patients with various pulmonary inflammations such as acute pneumonia, chronic bronchitis, bronchial asthma, especially in cases with mucoid impaction, and idiopathic interstitial pneumonia [6–9]. In our case, after 31 months, transient and slight re-elevation of serum CEA was seen, accompanied by the elevation of serum KL-6 and SP-D. CT examination at this time revealed minimal lung opacities, suggesting the presence of pneumonia in the left lower lobe, but no significant change in the irradiated area. This finding also suggested that the transient CEA elevation after SBRT was related to inflammatory changes of the lung in this case. The serum CEA level of smokers has been reported to be higher than that of nonsmokers [9]. CEA secretion from pulmonary epithelial cells has been considered to be influenced by smoking [10]. This patient was a heavy smoker, and serum CEA did not decrease to its normal level after SBRT. The elevation of the serum CEA level before SBRT may have been caused not only by the tumor but also by heavy smoking. 

Serum KL-6 and SP-D are useful serum markers of inflammatory lung diseases, typically interstitial pneumonia [11]. It has also been reported that KL-6 and SP-D can become elevated in some cases of adenocarcinoma [12]. In our case, however, serum KL-6 and SP-D levels were not elevated before SBRT. Also, serial changes of these serum marker levels were almost synchronous with the radiological change of the consolidation after SBRT and that following pneumonia in the left lower lung. Therefore, the changes in the serum KL-6 and SP-D levels after SBRT were considered to reflect the severity of the pulmonary inflammation rather than the progression of cancer.

## 4. Conclusion

We experienced a case of radiation fibrosis appearing as mass-like consolidation after SBRT with the elevation of serum CEA, which was difficult to distinguish from local recurrence. If it is difficult to make a definitive diagnosis by further examination including FDG-PET and histological examination, short-interval follow-up should be recommended.
